# Advanced Computing Methods for Impedance Plethysmography Data Processing

**DOI:** 10.3390/s22062095

**Published:** 2022-03-08

**Authors:** Volodymyr Khoma, Halyna Kenyo, Aleksandra Kawala-Sterniuk

**Affiliations:** 1 Faculty of Electrical Engineering, Automatic Control and Informatics, Opole University of Technology, 45-758 Opole, Poland; 2 Institute of Computer Technologies, Automation and Metrology, Lviv Polytechnic National University, 79000 Lviv, Ukraine; halyna.kenyo@gmail.com

**Keywords:** digital signal processing, analysis of rheographic signals, base impedance compensation, rheogram characteristic points, impedance plethysmography, avanced computing methods

## Abstract

In this paper we are introducing innovative solutions applied in impedance plethysmography concerning improvement of the rheagraph characteristics and the efficiency increase of the developing rheograms using computer methods. The described methods have been developed in order to ensure the stability of parameters and to extend the functionality of the rheographic system based on digital signal processing, which applies to the compensation of the base resistance with a digital potentiometer, digital synthesis of quadrature excitation signals and the performance of digital synchronous detection. The emphasis was put on methods for determination of hemodynamic parameters by computer processing of the rheograms. As a result–three methods for respiratory artifacts elimination have been proposed: based on the discrete cosine transform, the discrete wavelet transform and the approximation of the zero line with spline functions. Additionally, computer methods for physiological indicators determination, including those based on wavelet decomposition, were also proposed and described in this paper. The efficiency of various rheogram compression algorithms was tested, evaluated and presented in this work.

## 1. Introduction

This article aims to present new principles of signal and data processing in rheographic systems based on modern digital devices and computer methods. The implementation of these principles will make it possible to increase the potential of the rheographic method.

Rheography is another term for impedance plethysmography, and is a very important method applied for diagnostics of the cardiovascular system, with numerous advantages such as among the others [1,2]:

- non-invasiveness,
- harmlessness to the patient,
- high informativeness.

Based on the processing of rheographic data, it is possible to examine the state of central and regional hemodynamics, determine a number of physiological parameters, including ejection, blood volume per minute, and total peripheral resistance.

In medical diagnostics, methods of recording rheograms of any organ of the human body have been developed [3,4,5]:

- heart (rheocardiogram),
- brain (rheoencephalogram),
- great vessels,
- liver,
- lungs,
- limbs, etc. ...

Blood flow, condition of the vascular system, etc. This information significantly complements the results, in particular electrographic tests in the diagnosis of cardiovascular diseases and other pathologies, so often rheography is used in conjunction with ECG, EEG, etc. [6].

This work describes new methods of processing rheographic data in order to reject respiratory artifacts. A procedure for identifying characteristic points of the rheogram on the basis of the wavelet transformation has been proposed in this paper, as its advantages include formalizing the identification process and signal distortions affecting invariability. The results of the analysis of the contrasting effectiveness of the feedback signal compression algorithms are presented.

## 2. Materials and Methods

Currently, the rheography method is considered very promising and is widely used in various fields of clinical diagnostics and in physiological research [7].

The rheogram is a curve that corresponds to the dependence of the resistance of the tested system or human limb in the time domain $R_{\Delta }\left(t\right)$, and the devices used to record it are called rheographs [6,7].

The human body tissues are capable of conducting an electric current. The main charge carriers in them are ions. Blood as a distinct electrolyte is characterized by the lowest resistance (ρ≈ 1.5 Ohm · m), while the other tissues are much higher—skin (ρ≈ 5.5 Ohm · m), fat (ρ≈ 15 Ohm · cm), bone tissue (ρ≈ 150 Ohm · m) [8].

The resistance of the examined area of the body *R* should change with time with heartbeats, because the blood supply to the organ changes during them. Measurement of the body resistance to direct current is difficult due to the resulting tissue polarization and the appearance of additional charges on the electrodes. For these reasons, medical rheography uses an alternating excitation current with a frequency of about 100 [kHz], where the main component of bioimpedance is active resistance *R*, while the share of the $X_{C}$ reactance is low [8,9].

The variable component of the $R_{\Delta }$ resistance caused by the pulsation of the blood flow is only 0.05÷0.1% of the base resistance $R_{O}$ of the examined body area. In the scale of absolute values, the range of the measured impedance variation ΔR is only 0.05÷0.5 Ω [9]. The basis of the mathematical model describing the relationship between the change in volume δV due to blood pulsation and the change in the $R_{\Delta }$ resistance caused by the Nyboer Equation (1), [1]:

(1) $$R_{\Delta }=\frac{\Delta V}{\rho {\left(\frac{L}{R_{O}}\right)}^{2}},$$

where:

- ρ–blood resistivity, its approximate value;
- *L*–length of the tested body area.

The method of construction of the rheograph measuring track has a significant impact on the reliability of the results of rheographic tests. Usually in modern rheographs the so-called tetrapolar current system of 4 electrodes, ensuring the mitigation of the parasitic influence of the contact impedance (electrode and tissue contact) and a more even distribution of the current density with the appropriate distance between the application and receiving electrodes [8,10,11].

Traditional, conventional rheographic systems ensure the registration of three quantities [9,10,12]:

- the base impedance $Z_{O}$,
- the rheograms $Z_{\Delta }\left(t\right)$,
- its derivative $Z_{D}\left(t\right)=dZ_{\Delta }/dt$.

And these work in a following way: the examined area of the patient’s body through the so-called the application electrodes are given an excitation current with the frequency $F_{O}$. With the help of two receiving electrodes (located between the application electrodes), the potential difference ΔU is measured, which is proportional to the resistance of the tested organ. The voltage ΔU is amplified by the selective differential amplifier and fed to the low-pass filter in its rectified form. Since the cutoff frequency of the filter is lower than $F_{O}$, a signal component is formed at its output proportional to the base impedance $Z_{O}$.

Further in the processing chain, the high-pass filter eliminates the constant component in this signal, and the amplifier amplifies its variable component 1000 times, which reflects the pulsation (fluctuation) of the impedance $Z_{\Delta }\left(t\right)$ caused by filling the patient’s body with blood. The registration of this quantity over time is the ΔR(t) rheogram.

By analyzing the characteristic points of the rheogram, basic hemodynamic parameters are calculated, such as among the others those listed below:

- stroke volume,
- cardiac output,
- general resistance of the peripheral vascular system,
- pulse,
- pre-ejection period,
- time of blood ejection from the left ventricle,
- minute capacity.

Determination of the characteristic points is easier while having the first derivative of the rheographic signal $R_{D}\left(t\right)$ which is obtained by the differentiator. Of course, the signals from the outputs of each of the three channels can also be digitized with an analog-to-digital converter, allowing further computer processing. The analysis of the technical properties of rheographs showed some limitations regarding the range of measurement of the base resistance. Diagnostics such as rheovasography in the study of blood flow to the extremities, rheoencephalography in the study of blood flow to the brain, rheohepatography in the study of blood flow in the liver, rheopulmonography in the study of blood flow in the lungs, requires of the measuring range in the part of the base resistance—up to 1000 Ω [8]. In known rheographs, the base resistance measurement range is limited mainly by the values of 200 ÷ 250 Ω [5,9,11].

In order to extract the impedance of the variable $Z_{\Delta }\left(t\right)$ against the background of the dominant base impedance $Z_{O}$, the existing rheographs use low and high pass filters [4,9,13]. The implementation of analog high-pass filters with a very low cut-off frequency of 0.05–0.3 [Hz] to separate the proportional voltage $Z_{\Delta }\left(t\right)$ is based on high-capacity capacitors, and the instability of the filter parameters results in phase distortions of the registered rheograms. Another drawback of the measurement path structure of the known rheographs is that patient respiratory artifacts or electrode displacement, especially during training, may cause the measurement range to be exceeded for a long time because the filter time constant with cutoff frequency of 0.05 [Hz] is large. These factors limit the application areas of the rheographic test method, for example in mobile applications.

In this study, the authors presented an innovative solution based on base impedance compensation with a circuit containing an actuator in the form of a digital potentiometer. Moreover, in the proposed solution for the construction of the measuring circuit, a digital phase-sensitive detector was used, separating the negative influence of reactance. As a result, the waveform $R_{\Delta }\left(t\right)$ is recorded, not $Z_{\Delta }\left(t\right)$, which corresponds more closely to the mathematical Equation (1).

The credibility of the results of rheographic tests depends not only on the accuracy of recording $R_{\Delta }\left(t\right)$ waveforms with a rheograph, but also on the methods of developing the rheograms. These methods can be used to eliminate various kinds of distortions, primarily respiratory and movement artifacts. This leads to baseline drift in the course of the recorded reogram. This drift in turn causes errors in the determination of hemodynamics of the cardiovascular system.

To date, a number of different methods have been developed to remove distorting respiratory artifacts from the signal $Z_{\Delta }\left(t\right)$ and its derivative $Z_{D}\left(t\right)$. The work [14] proposes baseline drift compensation using an electronic circuit built on operational amplifiers and digital to analog converter, the [15,16] present an approach for artifact removal based on implementation of adaptive filters. Also, a number of works have been devoted to research on the effectiveness of using wavelet transform [17,18,19,20] in order to reduce noise from rheograms. However, the implementation of wavelet transform (WT) is quite complex in terms of computation. Also the block structure of the WT, and thus introduces delays in the registration of the reogram.

In this study, a quick and computationally simple method based on the baseline drift approximation by a spline function was proposed to mitigate the impact of baseline drift. In addition, a comparative analysis of the effectiveness of the elimination of respiratory artifacts by the Spline method with the method based on the Discrete Cosine Transform and Discrete Wavelet Transform (DWT) was performed.

An important feature of diagnostic and monitoring systems, including impedance plethysmography, is the possibility of computer-aided and intelligent analysis of the obtained data. In order to reliably estimate such important indicators of the central and regional hemodynamics of the vascular system as Stroke Volume, Cardiac Output, Total Peripheral Resistance and others, correct identification of characteristic points on the recorded rheogram is required. These points, like the set of PQRST points in the ECG signal, are associated with various physiological events in the heart cycle.

In numerous studies wavelet transform were used in order to detect characteristic points. A common feature of these methods is the need for synchronisation of the analyzed rheogram with the ECG signals, echocardiographic or phonocardiogram [21,22,23].

In case of the impedance plethysmography systems used for long-term monitoring, a significant amount of data is collected and there is not only a need for fast real-time analysis, but also effective instruments for creating and storing rheogram records. Therefore, it is also important to test the effectiveness of different methods of signal compression of impedance plethysmography as specific time courses. The selection of the most effective compression methods will allow to reduce the volume of the archives of the recorded reographic runs, which are the primary documentation of the experiments and storage for the obtained test results.

## 3. Implementation of New Digital Signal Processing Methods for the Rheographic Systems’ Performance Improvement

For the purpose of the variable component of the ΔR impedance against the background of the dominant base resistance $R_{O}$ in the existing rheographs, the existing rheographs use low and high pass filters [4,9,10]. The implementation of analog high-pass filters with a very low cut-off frequency of 0.05÷0.3 [Hz] to separate the proportional voltage ΔR(t) is based on large-capacity capacitors, and instability of the filter parameters results in phase distortions of the recorded rheograms. Another drawback of the track design of known rheographs is that patient respiratory artifacts or electrode displacement may cause the measuring range to exceed the measuring range for an extended period of time because the filter time constant with cutoff frequency of 0.05 [Hz] is large. These factors limit the areas of application of the rheographic research method.

In order to calculate the variable component of the impedance ΔR regarding the background of the dominant base resistance $R_{O}$, the existing rheographs the analogue high-pass filters with a very low cut-off frequency of 0.05÷0.3 [Hz] to separate the proportional voltage ΔR(t) can be applied, which is based on capacitors high capacity, and the instability of the filter parameters results in phase distortions of the recorded rheograms. Another drawback of the track design of known rheographs is that patient respiratory artifacts or electrode displacement may cause the measuring range to be exceeded for a long time because the filter time constant with cutoff frequency of 0.05 [Hz] is large. These factors limit the areas of application of the rheographic research method.

The accuracy of rheographic examinations depends not only on the technical parameters of the rheographs, but also on the methods of developing the rheograms. These methods can be used to eliminate distortions, mainly caused by the breath, as well as to intelligently analyze rheograms to obtain many medical indicators [13,24,25,26,27]. It is also important to test the effectiveness of various compression methods, which will make it possible to reduce the volume of archives of the recorded rheographic runs, which are the primary document of the test result.

This paper aims to present new principles of signal and data processing in rheographic systems based on modern digital devices and computer methods. The implementation of these principles will enable to increase the potential of the rheographic method.

Digital signal processing technologies can be used to improve the technical and operational characteristics of rheographs and, above all, to increase stability, accuracy and flexibility [6,8,27,28,29].

**Figure 1:**
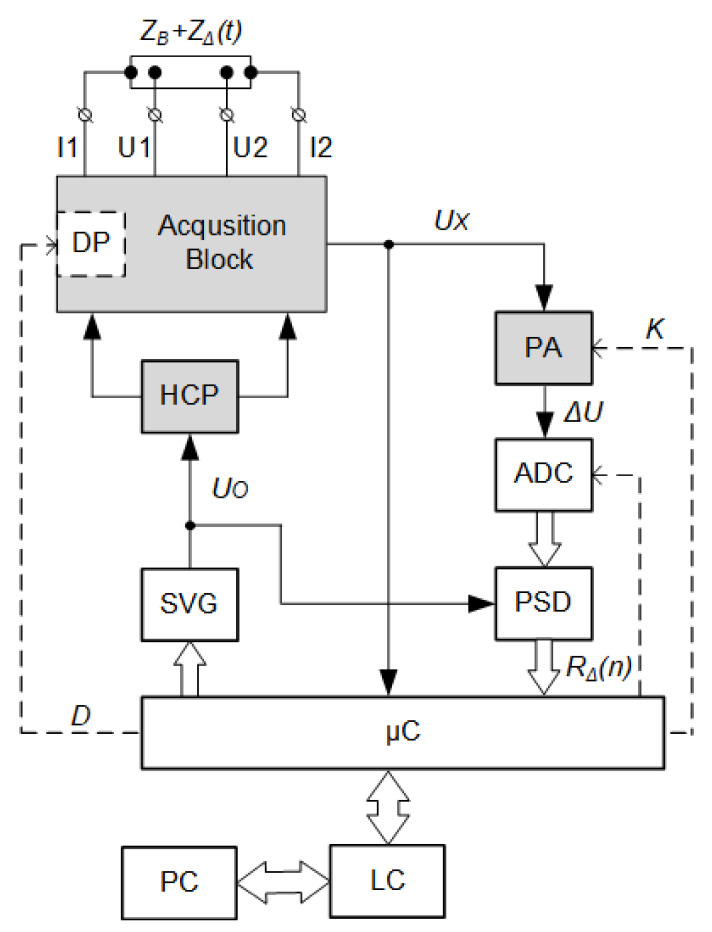
The structure of the digital rheograph (analog blocks in gray).

Figure 1 shows an innovative method of constructing a rheographic system, in which, unlike the known solutions, the number of analog structural elements has been minimized to three. The characteristic features of the proposed system include: a digitally controlled current source, base resistance compensation with a digital potentiometer and digital phase-sensitive detection based on an innovative solution [30].

The proposed digital rheographic system works in a way, that the excitation current $I_{O}$ fed from the Hawlend current pump (HCP) output to the application electrodes $I_{1}$ and $I_{2}$ is controlled by a digital sinusoidal voltage generator (SVG). This generator, built on the principle of Digital Direct Synthesis (DDS), forms a sinusoidal voltage $U_{O}$ with the frequency $F_{O}$ [30,31].

The potential difference is picked up from the surface of the patient’s body by means of the receiving electrodes $U_{1}$ and $U_{2}$ (2):

(2) $$\Delta U=U_{1}-U_{2}=Z_{\Delta }\times I_{O},$$

which is proportional only to the variable component of impedance $Z_{\Delta }$. For this purpose, the $Z_{O}$ base impedance compensation was applied in the acquisition block by means of a digital potentiometer (DP)—trimmer [32].

In the process of this compensation, the microcontroller (μC) plays a key role, because using the built-in analog-to-digital converter (ADC) it measures the voltage corresponding to the base impedance value, and then, after appropriate calculations, introduces a *D* code into the trimmer that compensates for the voltage drop on the tested bioimpedance and trimmer. After compensation, the value of the voltage $U_{X}$ at the output of the acquisition block tends to zero, so it is possible to amplify very small voltage fluctuations ΔU in the programmable amplifier (PWN), containing information useful for rheographic research. This method of base impedance compensation replaces the need for an analog high-pass filter with a very low cut-off frequency. The value of the amplification factor *K* of the amplifier is generated by a microcontroller in a way that allows to adjust the current values of the acquisition block voltage to the measuring range of the analog-to-digital converter (ADC).

From the analog-to-digital (ADC) converter, the instantaneous voltage values ΔU(n) are sent to the digital phase-sensitive detector (PSD) built according to an innovative solution [33]. Synchronous detection, performed with phase-sensitive detectors, enables the processing of very low-level signals [34]. In known impedance plethysmographs [35], the phase-sensitive detector is implemented on analog circuits (multiplier AD9837 and a low pass filter). The proposed structure of the rheographic system uses a digital phase-sensitive detector, built according to an innovative, patented solution [33]. The reference input of this detector is connected to the SVG synthesizer, which results in achieving detection of only the active component $R_{\Delta }\left(n\right)$ of bioimpedance. This allows the excitation current frequency range to be extended without the fear of a negative influence of the reactance—the reactive part $Z_{\Delta }$, which is not related to the blood flow. An additional advantage of the use of phase-sensitive detection is the combination of the flexibility of $F_{O}$ frequency tuning while maintaining high selectivity, ensuring an increase in the signal/noise ratio without the use of additional filters [33,36,37].

Controlling the phase of the synthesized signal is also important for the construction of rheographic systems. It is possible to use two DDS synthesizers operating at the same clock frequency, but with the phases of the exciting currents set quadrature. This is especially important when rheographic examinations concern symmetrical parts of the patient’s body. In order to obtain correct comparable results, tests should be carried out on one and the same frequency. In this case, there is distortion due to the interactions of the channels. Synchronous signal detection allows to eliminate channel interference even at one frequency of orthogonal measurement channels working in parallel.

In the structure of the digital rheographic system, the microcontroller performs a number of functions [30]:

- signal conditioning (equalization of the amplitude-frequency characteristics of the measuring path, digital signal filtering);
- representation of the measurement results (the time series corresponding to the rheograms, its first derivative and the base resistance);
- actuation of the trimmer resistance to compensate for the base impedance, therefore, an on-going compensation of the base resistance drift due to the displacement of the electrodes on the surface of the patient’s body is provided;
- setting the DDS synthesizer parameters for selecting the frequency and phase of the excitation current;
- control of an analog-to-digital converter (ADC) and programmable amplifier;
- communication via the level converter (LC) with a PC via the RS-232 interface.

## 4. Results and Discussion

This paper presents the results of a comparative analysis of the methods of processing recorded rheographic data aimed at:

- elimination of the respiratory artifacts;
- computing physiological indicators;
- compression of the rheographic wave forms.

**Figure 2:**
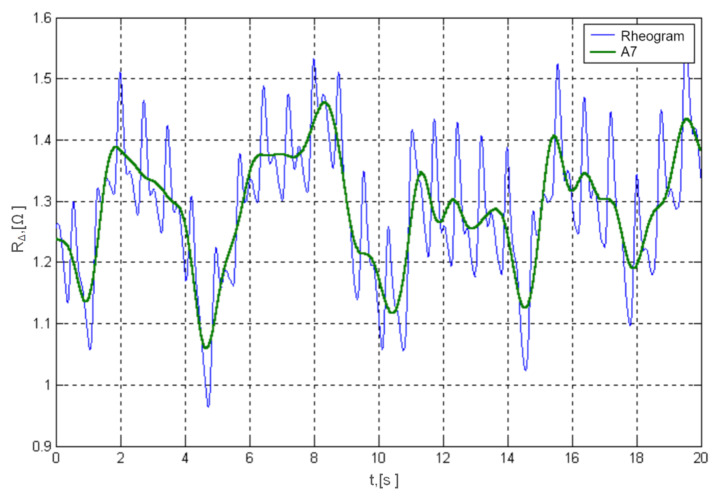
Rheogram fluctuations recorded in real conditions (blue line) and baseline drift approximation (green line).

Graph from the Figure 2 clearly shows the quasi-periodic fluctuations caused by the patient’s breathing, where there are two components in the rheographic signal [13]:

1. a useful rheographic signal to reflect fluctuations in blood supply to the organ or limb under test caused by heartbeat;
2. slow baseline drift due to patient breathing (jamming signal).

The signal of the baseline respiratory drift is a low frequency oscillation with a bandwidth of about 0.05–1.5 [Hz]. And the frequency range of the actual rheographic signal is within the range from 0.5 to 20 [Hz], [8,28]. As the frequency ranges of both components differ from patient to patient and depend to a large extent on the recording conditions of the rheogram (resting or dynamic loads), the use of filters with constant parameters is ineffective.

For the purpose of conditioning the rheograms, three methods to eliminate the baseline drift were investigated:

1. based on the Discrete Cosine Transform (DCT);
2. based on the discrete wavelet transform (DWT);
3. using a spline approximation (SA).

The research was carried out on a typical rheogram signal from the database which is part of the integrated “Rheonite“ information and diagnostic rheocomplex. A 12-bit analog-to-digital converter (ADC) with a sampling frequency of $F_{s}=250$ [Hz] was used to digitize the analog signal.

The discrete cosine transform turned out to be very convenient in practice because, in addition to eliminating the zero line drift, it allows filtering out high-frequency noise and obtaining the waveform of the first derivative of the rheogram. The DCT method is computationally efficient because there are fast algorithms for its calculation and, moreover, unlike the discrete Fourier transform, it does not lead to complex coefficients. The straight line and inverse DCT are described by the Equations (3) and (4):

(3) $$X\left(k\right)=w\left(k\right)\sum_{n=0}^{N-1}x\left(n\right)\times cos\left[\frac{\pi (2n+1)k}{2N}\right],$$

(4) $$x\left(n\right)=\sum_{k=0}^{N-1}w\left(k\right)\times X\left(k\right)\times cos\left[\frac{\pi (2n+1)k}{2N}\right],$$

where:

$w\left(k\right)=\left\{\begin{array}{c} 1/\sqrt{N},k=0\\ \sqrt{2/N},1\leq k\leq N-1. \end{array}\right.$

The noise elimination based on the discrete cosine transform includes:

- estimation of the cuto-ff frequency on the basis of the signal power spectral density analysis, in which the main energy of the $F_{BL}$ interfering signal is concentrated;
- computing the DCT coefficients that fall in the band from $F_{BL}$ to the maximum frequency of the $F_{R}$ rheogram;
- calculation of inverse DCT.

Taking into account the non-stationarity of the rheographic signal, the Welch method was chosen to estimate its spectrum. The length of the analyzed rheogram was 5000 samples, with a segment width of 1000 and an overlap of 500 samples. The data was smoothed with the use of Hann’s window.

Studies have shown (see inter alia: [30,38,39]) that for the algorithm to operate without noticeable distortions of the useful rheographic signal, the frequency resolution must be at least 0.1 [Hz], which corresponds to a segment length of 2500 samples. For example, for the signal shown in Figure 2, the cut-off frequency of the zero line is $F_{BL}\approx 0.95$ [Hz], and the maximum frequency of the useful rheogram is $F_{R}\approx 15$ [Hz]. Then the number of DCT coefficients that fall in this band and need to be calculated is 281.

The disadvantages of the discrete cosine transform include the block character of the algorithm and the inability to obtain information about the cutoff frequencies of the rheogram ($F_{BL}$ and $F_{R}$) directly from the DCT spectrum because it is not a smooth and statistically stable function.

One promising approach to eliminate the effects of respiratory artifacts is the use of the Discrete Wavelet Transform (DWT). The wavelet transform enables the time-frequency representation of non-stationary signals by decomposing the rheogram into approximation components (represented by the coefficients cAj) and detailing (represented by the coefficients cDj). It can be expected that at some level of the distribution the approximation component, containing information about the low frequency components of the signal, will be a good approximation of the baseline drift [26,40].

**Figure 3:**
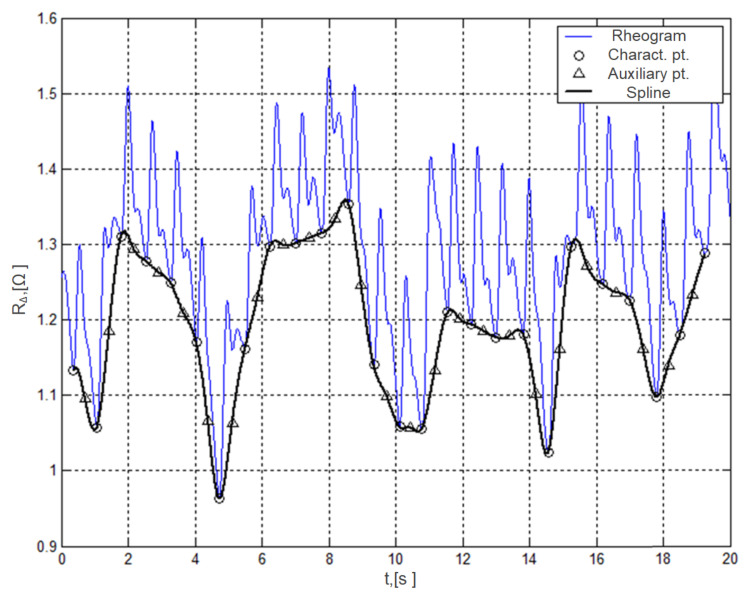
Fluctuations of the rheogram recorded in real conditions (blue line) and the baseline drift joint function approximation (black line).

The Daubechies 15 wavelet type was selected empirically and it was found that the seventh level of $A_{7}$ decomposition is optimal for the isolation of respiratory artifacts (Figure 3). The difference between the recorded output rheogram and the approximation curve represents a useful signal, i.e. the relevant course of the rheogram (see: Equation (5)):

(5) $$R=R_{\xi }-A_{7}.$$

The algorithm testing on real rheograms showed that the minimum size of the processed block should be 1500–2000 samples of the analyzed signal. It should be also noted that this algorithm does not require any a priori information about the spectrum of the disturbed rheogram.

The advantages of DWT include the possibility of combining the baseline drift approximation operation with cleaning the rheogram from other disturbances [40,41]. For wavelet decomposition, no additional information about the disturbance spectrum is required, and the wavelet transform itself works on real numbers. The disadvantages of the wavelet transform include the block structure of the algorithm causing delays of 5–10 s, the relative complexity of calculations and limited flexibility in the selection of parameters.

The use of spline functions to approximate the baseline drift allows for a departure from the block structure of the processed data, which is characteristic for the DCT and DWT methods. The main idea of the method is to select characteristic points on the rheogram that serve as nodes of the interpolation polynomial approximating respiratory artifacts. For the characteristic (reference) points, moments are selected when the differential rheogram has zero values, which also corresponds to the beginning of the rheocycle. It is possible to prevent occurrence of unnecessary oscillations and to increase the accuracy of the approximation by using additional interpolation nodes located in the middle of the line connecting adjacent characteristic points (Figure 3).

The carried out research showed that the 3rd degree spline function fully meets the requirements for the accuracy and efficiency of approximation. The advantage of a spline approximation is low computational complexity and minimal (in one cardiocycle) delay in processing the rheogram.

**Figure 4:**
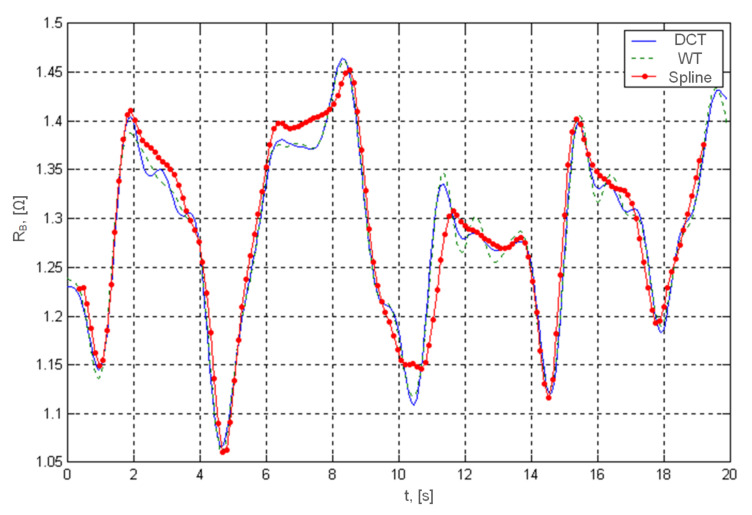
Approximation of the respiratory artifacts of a real rheosignal by different methods.

In order to better investigate the properties of the proposed algorithms, studies were carried out on a synthesized rheography-signal containing known heart rate oscillations and respiratory disturbances. The research showed that the baseline drift approximation methods using the wavelet transform and DCT give slightly more accurate results than the approximation by spline functions (Figure 4). However, working with real signals, in conditions of both heart rate variability and respiratory artifacts, the differences between the accuracy of these methods are blurred, and the main advantages of the method of approximation by glued functions are primarily low computational complexity.

Table 1 presents a comparison of the methods for the analysis and elimination of respiratory artifacts according to such features as:

- CE–computational efficiency;
- ID–introduced delay;
- EA–estimation accuracy;
- EN–elimination of higher frequencies noise;
- PA–preliminary frequency analysis.

**Table 1:** Comparative analysis of baseline drift suppression methods.

Analyzed Feature	Method		
	DWT	DCT	SA
CE	high	**middle**	**low**
ID, heartbeat	~10	~10	**one**
EA	**good**	**good**	**moderate**
EN	**Yes**	**Yes**	No
PA	**not required**	required	**not required**

The comparative analysis showed the advantages of the cohesive function approximation method, first of all, low computational complexity and minimal delay introduced during baseline drift suppression.

### 4.1. Computer Methods for Determination of Physiological Indicators

On the basis of the amplitude-time parameters of the rheogram according to the known formulas, the most frequently calculated indicators of hemodynamics are: Cardiac Output (*CO*), Stroke Volume (*SV*), Total Peripheral Resistance (TPR), Systemic Vascular Resistance (SVR) (see: Equations (6)–(8)) [7,40,42,43]:

(6) $$SV\left[ml\right]=\rho \frac{L^{2}}{R_{O}^{2}}\cdot T_{BE}\cdot {\left|\frac{dR_{\Delta }\left(t\right)}{dt}\right|}_{max}=\rho {\left(\frac{L}{R_{0}}\right)}^{2}T_{BE}A_{D},$$

(7) $$CO\left[l/min\right]=\frac{SV\times HR}{1000},$$

(8) $$CI\left[l\cdot min^{-1}\cdot m^{-2}\right]=\frac{CO}{S},$$

where:

ρ—specific resistance of blood, approximately 150 [Ω /cm];

HR—heart rate (number of heart beats per minute);

CI—Cardiac Index;

RΔ(t)—the impedance change during systole [Ω];

$A_{D}={\left|\frac{dR_{\Delta }\left(t\right)}{dt}\right|}_{max}$—the maximum of the first rheogram derivative with respect to time [Ω/sec];

*L*—distance between the measuring electrodes [cm];

$R_{O}$—base resistance (Ohm);

$A_{D}$—the first derivative of the rheogram amplitude (Ohm/s);

$T_{BE}$—blood ejection period, which is determined from the beginning of the rheocycle to the first minimum of the rheogram, following the maximum of its derivative (see Figure 5).

As one can see, the stroke volume is a key parameter as it appears in the definition formulas of other indicators. Since it is assumed that in the measurement process the distance L between the electrodes and the base resistance $R_{O}$ are constant, two quantities should be measured to determine the stroke volume—$A_{D}$ and $T_{BE}$.

Traditionally, the contour analysis of the rheogram and its first derivative is used to calculate the physiological indices of the circulatory system, which includes a search for characteristic points on these curves. First of all, these are the three points (Figure 5):

- B–the point directly related to the beginning of ejection of blood by the left ventricle and corresponds to the zero crossing point by rheogram derivative;
- E–point directly related to a moment of the maximal velocity of the blood ejection during systole and located on the high position of the curve reflecting the maximal speed of the change in the impedance;
- X–point associated with the aortic valve closure, which is below the baseline on the low position of the rheogram derivative.

Pre-processing of the rheogram with the conventional methods includes filtering, differentiation, smoothing, calculation of threshold values, selection of intervals with characteristic points [22,42,44]. However, the digital methods of differentiating a rheogram applied in order to find its first derivative are particularly sensitive to disturbance and noise. To preserve the shape and timing of the useful signal, digital filters with a high-order finite impulse response should be used, providing a linear phase-frequency response. The algorithms for recognizing characteristic points, based on the sequential search for extremes in open time intervals, lead to a program structure with a large number of branches and conditional operators, which significantly increases the processing time. Therefore, it is especially important to develop methods that would minimize the complexity and time of signal processing and would allow for the elimination of the time-consuming manual marking of characteristic points [39,45].

Therefore, it is very interesting to define the diagnostic parameters of the frequency domain rheogram. For this purpose, it is necessary to establish the relationship between the amplitude-time parameters important for diagnostics and the spectral composition of the rheogram and its derivative, and to evaluate the accuracy of the results. Two methods of automatic determination of hemodynamics indices were investigated for this study purpose—the discrete cosine transform (DCT) and the wavelet transform (WT).

The research was carried out on three rheograms recorded under rest conditions, during and after physical activity for 20 s. The resulting raw X(n) rheograms, which are an overlay of information signal and noise and artifacts, were processed by a Savitsky-Golay third-order smoothing filter with a frame length of 45 samples. The filtered R(n) rheograms were differentiated by $R_{D}\left(n\right)$ and divided into rheocycles of *N* length sample. The total number of rheocycles was 210. In each of the *i*-th rheocycle $R_{Di}\left(n\right)$ the characteristic points $A_{Di}$ and $T_{BEi}$ and the corresponding sample numbers were found by analyzing the contours—$n_{Ai}$ and $n_{Ti}$. So it had to be established how to compute the characteristic points $A_{Di}$ and $T_{BEi}$ from the DCT coefficients.

The carried out research has shown that for the spectral representation of the rheogram and its derivative with an error of no more than 1%, the minimum number of DCT coefficients is 9 and 16, respectively. Determining the characteristic points using the DCT method includes the three following stages:

1. determination of the spectral coefficients $C_{i}\left(k\right)$ on the basis of the DCT analysis of the raw rheogram $X_{i}\left(n\right)$;
2. synthesis by the inverse DCT of the relevant rheogram $R_{i}\left(n\right)$ as well as its derivative $R_{Di}\left(n\right)$ by formal differentiation of the DCT series;
3. localization and estimation of the $A_{Di}$ and $T_{BEi}$ characteristic points.

A comparative analysis of the error in determination of the stroke volume according to the above method with the contour analysis showed that the systematic error in determining the SV is equal to 1%, and the standard deviation is 2.6%, which, compared to the accuracy of the rheographic method, fully meets the needs of practice.

**Figure 6:**
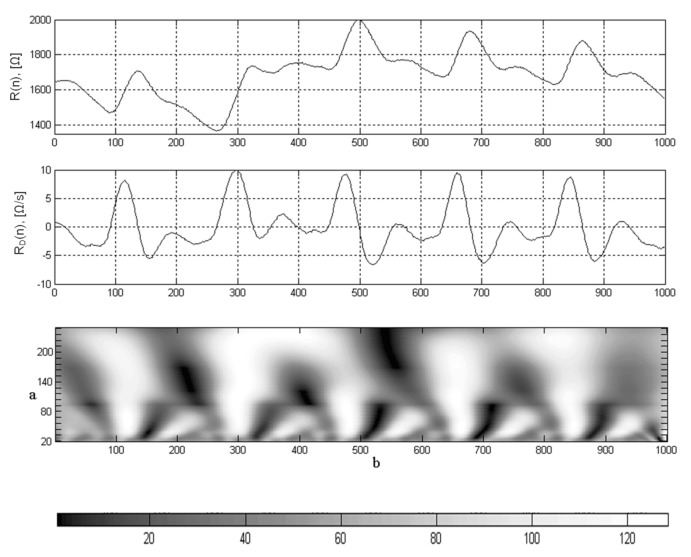
Waveform of the raw rheogram (**top**), its first derivative (**middle**), wavelet scalogram of derivative rheogram (**bottom**); along the horizontal axis—sample numbers.

A serious limitation of the DCT method in identification of the characteristic points of the rheograms is the lack of information about the temporal characteristics of the signal, which complicates the task of locating these points on the time axis. The wavelet transform allows to solve this problem due to the peculiarity of interpreting the signal on the time-frequency plane. As a result of the wavelet decomposition, the coefficients C(a,b) reflect the components of the rheogram in a given time interval and in the frequency band. As a result of wavelet decomposition, the coefficients C(a,b) reflect the components of the rheogram in a specific frequency band and time interval–parameters *a* and *b*, respectively (Figure 6).

The Coiflet5 wavelet was selected to detect the characteristic points of the rheogram. The central frequency of this wavelet is 0.6897 [Hz], so for $F_{S}=250$ [Hz] in the range of coefficients a=250 and a=20, the range of center frequencies is from 0.6897 to 8.6 [Hz].

**Figure 7:**
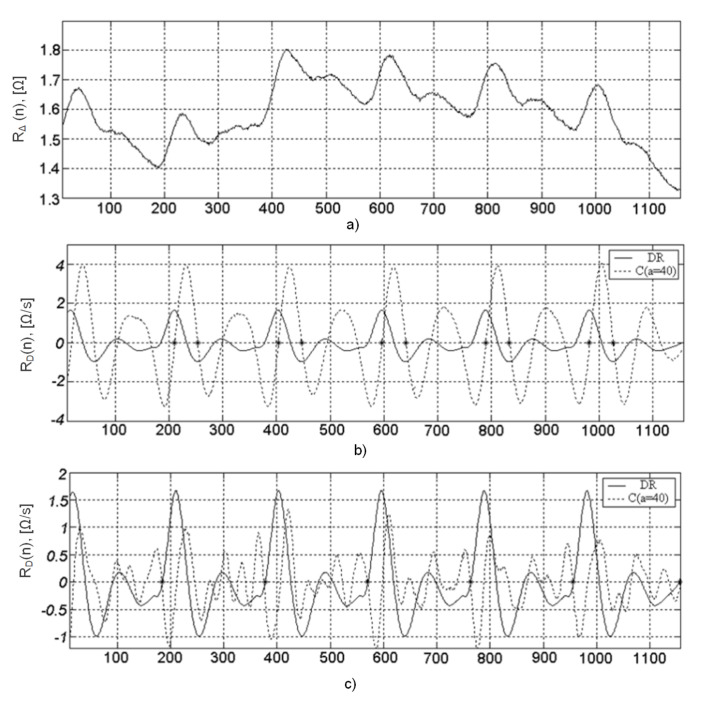
Recognizing the characteristic points of the rheogramme; along the horizontal axis—sample numbers.

After conducting preliminary tests, it was found that for the analysis of the differential rheogram maxima corresponding to the fastest signal change and correspondingly containing high frequencies, a=21 (4.9 [Hz]) should be chosen, and the minima—a=52 (3.3 [Hz]). Thus, when analyzing the maximums of the C(35,b) coefficients (Figure 7a), it is possible to determine the points of the maximum blood filling and in accordance with the C(52,b) minima (Figure 7b)—the ejection period (Figure 7).

**Figure 8:**
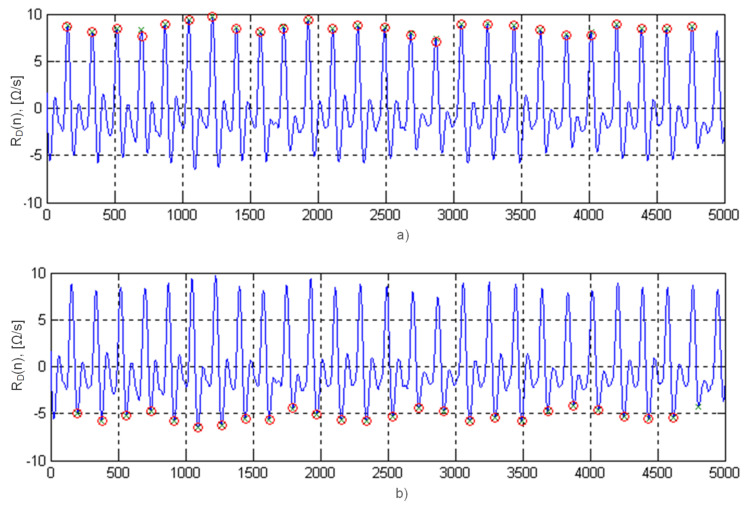
Recognition of the rheogram characteristic points along the horizontal axis—sample numbers.

Thus, by analyzing the moments of crossing baseline by the coefficients of the wavelet transform C(52,b) from negative to positive values, it is possible to determine the points E(i) of the maximum blood filling (Figure 8a), and in opposite direction (Figure 8b)—points X(i). At the instant of time E(i), they read the values of an important diagnostic value ADi from the rheogram derivative curve, i.e., the maximum blood ejection velocity [42].

The characteristic points B(i) can be located after the coefficient C(35,b) of the wavelet transform (Figure 7c). The location of points B(i) and X(i) on the timeline allows the calculation of the blood ejection period $T_{BEi}=X\left(i\right)-B\left(i\right)$.

Characteristic points of the rheograms determined by the contour analysis using the previous isoline drift elimination procedures, filtering, smoothing, differentiation, practically coincide with the points determined by the appropriate wavelet transform coefficients without performing any re-signal conditioning operation (Table 2 presents the data obtained from the first 10 rheocycles). This is due to the fact that the analysis is carried out in those frequency ranges where the influence of artifacts and noise is minimized.

The comparison of the method of automatic recognition of characteristic points based on the wavelet transform with the traditional contour analysis showed no systematic error in the calculation of the stroke volume (after averaging on the entire database of each patient), and the value of the consolidated mean square error was 1.8%.

### 4.2. Rheographic Waveforms Compression Methods

The development of efficient rheographic data compression algorithms is an important aspect of the improvement of existing rheographic monitoring and diagnostic systems, as well as a condition for the development of portable devices with acceptable requirements for built-in memory and power consumption [43]. Looking at the rheograms as specific time series, the authors examined the effectiveness of compression algorithms that are used in various fields to compress signals and data. This paper presents the results of research on the effectiveness of rheographic data compression with the use of algorithms belonging to three below listed classes:

1. direct compression of signals,
2. compression based on transforms,
3. and parametric compression.

Among the algorithms representing direct compression, Huffman statistical coding (HC), aperture coding (AZTEC—Amplitude Zone Time Epoch Coding), delta modulation (DM) and differential pulse code modulation (DPCM) were investigated. Trasnform-based compression algorithms are represented by Walsh transform (TW), discrete cosine transform (DCT), Karunen-Loeve transform (TKL), and discrete wavelet transform (DWT). Parametric compression algorithms included short-term prediction (STP), long-term prediction (LTP), codebook (CB) and averaged rheocycles (AR).

The performance criteria are the compression ratio, the normalized mean square error of the signal reconstruction and the computational complexity of the algorithm. The characteristics of the methods were determined using the pre-selected optimal parameter values for each method.

Table 3 shows the results of the tests of the mentioned compression algorithms on real rheograms of various patients.

Based on the comparative analysis of the compression ratio and accuracy, it can be concluded that the most effective in relation to the rheographic data were the compression methods based on delta modulation and discrete cosine transform. For monitor rheographic systems where low computational complexity comes to the fore, the use of a compression algorithm based on the Walsh transform is promising.

## 5. Conclusions

Impedance plethysmography is an important technique of medical diagnosis and clinical monitoring. The functionality of this technique and the reliability of the results largely depend on the hardware support that ensures the registration of rheograms, and the algorithms that allow the computation of biomedical indicators through rheogram processing. Automation of such processing is hampered by physiological variability, which often contains useful diagnostic information, a large dynamic range, and a low level of useful signal against the background of artifacts. To solve such a complex problem, it is possible to use the computing power of a microcontroller built into the rheographic system, and a computer for processing and storing rheogram records. Therefore, the research results presented in the paper lie in two directions.

The first direction concerns the improvement of the rheographic system at the lower instrumentation level, which will ensure higher accuracy and reliability of the registered rheograms. This can be achieved through the proposed innovative structure of the rheographic system, which uses digital synthesis of quadrature probing stresses and the implementation of synchronous detection in digital form.

The use of digital synthesizers of orthogonal signals increased the flexibility of choosing the frequency of the probing current and made it possible to exactly maintain their quadrature. As a result, this allows you to expand the diagnostic capabilities of rheographic studies due to the parallel registration of rheograms from symmetrical parts of the body at different frequencies. An important feature of the rheographic measuring path is also the use of a digital phase-sensitive detector, implemented according to an innovative solution. The main advantages of using phase-sensitive detection in rheographic systems are increased noise immunity and separation of resistance ripples (rather than impedance, as in existing rheographs).

Another important feature of the proposed structure of the rheograph is the use of a programmable digital potentiometer to compensate for the base resistance of the $R_{O}$. This made it possible to eliminate the masking effect of the dominant $R_{O}$ component and increase the sensitivity of the measuring path to the useful signal $R_{\Delta }\left(t\right)$ without the need to use an analog high-pass filter with a very low cutoff frequency.

The second direction concerns the highest level of the rheographic system, that is, methods for processing rheograms on the PC. In the case of long-term monitoring, a significant amount of data is collected and there is a need for fast, real-time analysis. Within the framework of this direction, methods for analyzing and suppressing the drift of the baseline and methods for automatically detecting and localizing the characteristic points of the rheogram have been proposed and studied.

The paper presents the results of a study of three methods for suppressing respiratory artifacts on rheographic records, the use of which improves the analysis and interpretation of rheograms. In addition to the often used wavelet decomposition, the application of the Discrete Cosine Transform have been also investigated. This transform also makes it possible to eliminate distortions caused by both respiratory artifacts and noise in the upper frequency band, but DCT, as known, is less demanding on computational resources compared with Wavelet Transform. The essence of the third method is based on the use of the cubic spline function for approximating the baseline drift. An assessment of the accuracy and computational complexity of the proposed methods for processing rheograms was carried out. Methods are identified that are not demanding on computing resources and provide acceptable accuracy, which is important for portable and monitoring rheographic systems.

The identification and determination of the parameters of the characteristic points of the rheograms is a necessary step in determining a number of important indicators of hemodynamics. The article presents a new solution regarding the formalization of the procedure of searching for characteristic points on the rheogram both in the frequency domain (Digital Cosine Transform) and in the time-frequency plane (Digital Wavelet Transform). The efficiency of the proposed methods, especially those based on DWT, has been confirmed by the example of blood stroke volume estimation.

Another important issue for computer diagnostic and monitoring systems is the recorded data archiving. The rheograms compression methods were tested on real rheograms, the use of which increases efficiency. The effectiveness of compression algorithms belonging to direct coding methods and based on orthogonal and parametric transforms was tested. It was found that according to the aggregated index “compression ratio/reconstruction error/computational complexity”, the algorithms based on the Walsh transform, delta modulation and discrete cosine transform should be preferred.

## Figures and Tables

**Figure 5:**
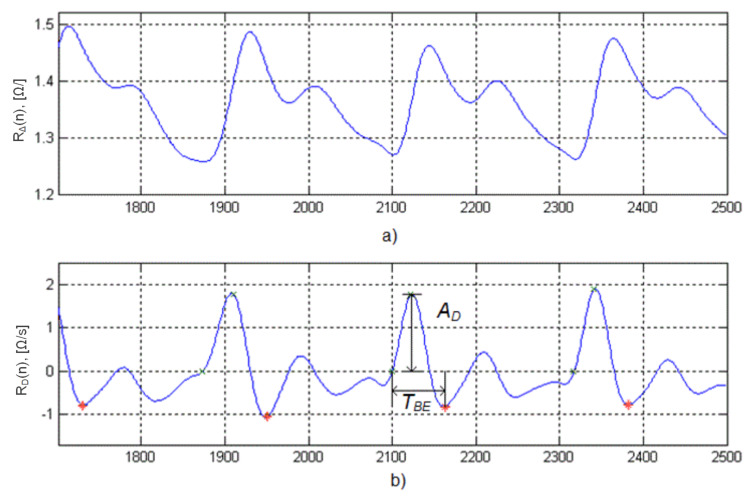
Rheocycle (**a**) and its derivative with characteristic points (**b**).

**Table 2:** Verification of the accuracy of the rheograms characteristic points determination by means of wavelet analysis.

Rheocycle No.	RDI Maxima Points		RDI Minima Points		WO Error −%
	Contour Analysis	Wavelet Analysis	Contour Analysis	Wavelet Analysis	
1	156	154	201	201	1.2
2	337	338	380	381	−1.0
3	521	520	563	564	−1.1
4	701	708	744	745	4.8
5	874	874	919	918	1.3
6	1050	1050	1094	1093	1.3
7	1224	1224	1270	1271	−1.4
8	1401	1400	1444	1445	−0.8
9	1578	1578	1621	1622	−1.2
10	1750	1746	1798	1795	6.6

**Table 3:** Comparison of the rheogram compression methods.

Method	Compression Ratio	Reconstruction Error −%	Computational Complexity
HC	1.3	–	Medium
AZTEC	4.8	3–4	Medium
DM	12	1.3	Medium
TW	4–6	1.5–2.5	Low
DCT	6–8	1.2–1.8	Medium
TKL	6–8	1–2.5	High
DWT	9–11	2–4	High
DPCM	6	1.3	Medium
STP	∼3.5	–	Medium
LTP	∼4.5	–	High
CB	5.5	–	High
AR	3.5	–	Medium

## Data Availability

Data available upon written request from the corresponding author.
